# HARNESSING THE HEALING POWER OF WESTERN AND NON-WESTERN HEALERS: IMPLICATIONS FOR SOCIAL WORK PRACTICE

**DOI:** 10.1093/geroni/igz038.1602

**Published:** 2019-11-08

**Authors:** Rashmi Gupta, Vijayan Pillai

**Affiliations:** 1 san francisco state university, san francisco, California, United States; 2 University of Texas at Arlington, Texas, United States

## Abstract

Non Western therapies have played a vital role in dealing with a variety of crisis such as physical, emotional and existential over the last few centuries (Ejaz, 2000). Since the introduction of Western medicine in India as early as the eighteenth century, the two systems of medicine have coexisted and influenced each other. Mutual recognition of the therapeutic values of the two systems was further bolstered when American medicine recognized the role of alternative therapies as viable option for healing in the early eighties. The purpose of this paper is to examine the roles two kinds of healers (western and non- western) play in the Asian Indian Hindu context. For this qualitative study, 30 physicians (trained in Western medicine) and 5 sadhus (holy men) were interviewed in the city of Allahabad, during the Kumbh Mela. Open ended questions ranged from: a) field of practice (physicians), sadhus (mystics), b) years of practice, c) number of patients/followers, d) treatment offered (physician –surgery or medications), sadhus (prayers, fasts, charitable donations), e) adverse reactions. In-depth interviews were recorded and transcribed by two researchers from Hindi to English and coded into themes. Results indicate that about a quarter of Western trained physicians not only sought assistance from the sadhus for their emotional/spiritual issues, but also referred patients with terminal disease. Besides individual consults, the mystics conduct lectures on climate change, on holistic diet, meditation, yoga practices, and healing. Social workers need to assess the value Hindu immigrant families in United States attach to Non-Western therapies.

